# Stammering and Stuttering

**Published:** 1870-07

**Authors:** 


Stammering and Stuttering.—Dr. Abbotts Smith says, a large number of cases of stammering and stuttering can be cured; and of the remainder, nearly all may be relieved by the treatment which he indicates. He maintains that no one need be considered as hopeless, except those belonging to the small class dependent on malformation, or deficiency of the organs of speech. He instances, as causes of stammering and stuttering, (1) Too great eagerness in speaking; (2) Speaking with the chest only partially filled with air, which gives rise to sucking in the breath while speaking; (3) Malposition of the tongue; and (4) Speaking too low and indistinctly.

